# Patient safety culture perceptions among dentists in the eastern region of Saudi Arabia

**DOI:** 10.1186/s12903-024-04610-1

**Published:** 2024-07-21

**Authors:** Khalifa S. Al-Khalifa, Suliman Y. Shahin, Mishali AlSharief, Amal Asiri, Yousef AlYousef, Muhammad Nazir

**Affiliations:** 1 Department of Preventive Dental Sciences, College of Dentistry, Imam Abdulrahman Bin Faisal University, Dammam, 31441 Saudi Arabia; 2 Department of Dental Education, College of Dentistry, Imam Abdulrahman Bin Faisal University, Dammam, 31441 Saudi Arabia

**Keywords:** Patient safety, Health services, Dentistry, Health care, Clinical safety, Questionnaire

## Abstract

**Objective:**

Safe patient care can help reduce treatment costs, morbidity, and mortality. This study aimed to assess dentists’ perceptions of patient safety culture and related factors in the Eastern region of Saudi Arabia.

**Methods:**

This cross-sectional study used a sample of 271 dental professionals working in private and public dental hospitals and clinics in the Eastern region of Saudi Arabia. The Safety Attitude Questionnaire (SAQ), a validated tool consisting of 36 items on a 5-point Likert scale, was used to assess dentists’ perceptions of patient safety culture. The score of SAQ ranges from 0 to 100 and a cut-off ≥ 75 is considered a positive attitude toward patient safety culture.

**Results:**

There were 53.9% males and 46.1% females in the study with a mean age of 35.56 ± 6.87 years. Almost half of the participants (52%) attended a course on patient safety and 22.1% experienced medical error in the last month. The mean score of the SAQ of the sample was 65.14 ± 13.03 and the patient safety score was significantly related to the marital status (*P* = 0.041), attendance of patient safety course (*P* < 0.001), and experience of medical error (*P* = 0.008). The highest mean score (73.27 ± 20.11) was for the job satisfaction domain, followed by the safety climate domain (67.69 ± 16.68), and working conditions domain (66.51 ± 20.43). About one-quarter of the participants (22.5%) demonstrated positive attitudes toward patient safety culture. Multiple logistic regression analysis showed that dental professionals who attended a patient safety course were 4.64 times more likely to demonstrate positive attitudes toward patient safety than those who did not attend a course (*P* < 0.001).

**Conclusion:**

This study showed that patient safety culture was significantly related to the attendance of safety courses, marital status, and experiencing medical error. About one out of four dental professionals demonstrated a positive attitude towards patient safety culture which was significantly associated with the attendance of the safety course.

## Introduction

According to the World Health Organization, patient safety is defined as “the prevention of errors and adverse effects to patients associated with health care” [1]. While, patient safety culture is “the product of individual and group values, attitudes, perceptions, competencies, and patterns of behavior that determine the commitment to, and the style and proficiency of, an organization’s health and safety management” [2]. Patient safety culture is a multifactorial phenomenon and is affected by factors such as safety climate, morale, staffing, teamwork, supervision, and management support [3]. 

Accidents, errors, and complications are adverse events that can be prevented by enhancing patient safety culture [4]. In a prior research, 15,548 patient records from 26 hospitals across eight nations were examined. It was discovered that 8.2% of the records included at least one adverse event, 83% of which could have been prevented, and 30% of which were linked to patient death [5]. Medical errors account for 44,000 to 98,000 deaths of patients each year in the United States (U.S.) [6]. Economically, preventable adverse events cost $17 billion to $29 billion each year in the U.S. [7].

In dentistry, there are limited data regarding adverse events because such events are less severe in the dental profession than in the medical profession, dental practice being largely private (fear of losing profit in case of reporting adverse event), lack of generalized culture related to patient safety in dental practice, and difficulty in collecting data from widely distributed dental offices [8, 9]. For instance, unlike large hospitals or group dental practices, 80% of dentists work as a solo dentist in a dental clinic in the U.S. [10]. In addition, a lack of incident reporting systems in dental practice may contribute to lower reporting of adverse events in dentistry than in the medical field [11]. Nevertheless, adverse events are most common in implant dentistry, endodontics, and oral surgery, and up to 44.3% of adverse events occur due to predictable and preventable errors in dental clinics [12]. The analysis of 2012 patient safety incident reports in dental specialties showed that 36% of incidents were administrative errors, 10% injury, 6% medical emergency, 4% inhalation/ingestion, 4% adverse reaction, and 2% wrong site extraction [9]. 

Patient safety is an ethical obligation of dental professionals to ensure the quality of care for their patients. Improved patient safety is related to a better quality of patient care, reduced clinical problems, less legal claims, and greater legal security for dental professionals [13]. In dental schools and hospitals across the U.S., several studies assessed the patient safety culture among dental students, teachers, staff, and administrators [14–16]. Recently, a study reported patient safety culture among dentists, dental assistants, and supervisors from 20 dentistry departments in hospitals and 40 dental offices in China [17]. 

Saudi Arabia has been actively pursuing efforts to ensure quality improvements in healthcare systems [18]. In two different studies, dental students in Riyadh were asked about their awareness and attitudes towards patient safety. Interestingly, the perception of patient safety culture among dental students was low in both studies. As anticipated, perception should improve with further clinical training [19, 20]. However, there is little evidence regarding the awareness and attitude of dental professionals toward patient safety culture in Saudi Arabia. The investigation of the factors related to patient safety culture will help strengthen the initiatives to prevent adverse events and improve patient safety and quality of patient care in dental practice. This study aimed to assess dentists’ perceptions of patient safety culture and related factors in the Eastern region of Saudi Arabia.

## Methods

### Study design and population

This cross-sectional study was conducted in public and private hospital and dental clinics in the Eastern Province of Saudi Arabia. Dental professionals (general practitioner (GP), specialists, consultants) were included in the study from Dammam, Khobar, Dhahran, Alahsaa, Jubail, and Qatif cities. The sample size of dental professionals for the study (*N* = 334) was calculated based on the assumptions of 5% margin of error, 95% confidence level, the population of dentists in assigned cities was estimated to be *N* ≈ 2500, according to data from the General Directorate of Health Affairs in the Eastern region, and 50% response distribution. Through visits to the clinics, data were gathered, and non-probability convenience sampling was used to choose research participants. Dental practitioners who had given written informed permission and had been in practice in the province for the previous six months were eligible to take part.

### Instrument

Literature shows the use of eleven instruments for the evaluation of the safety culture in healthcare organizations [21]. In dentistry, “Hospital Survey on Patient Safety Culture” was used by Leon et al. to assess patient safety culture in seven American dental schools [14]. Later, Romani et al. and Yansane et al. used “Medical Office Survey on Patient Safety Culture” (MOSOPS) instrument to observe patient safety culture in various dental institutions in the U.S [15, 16]. 

In Saudi Arabia, the investigation of patient safety culture among female dental students from two dental colleges was carried out by Al-Surimi et al. using the Safety Attitude Questionnaire” (SAQ) [20]. Recently, a study of dental care providers in China also used the SAQ instrument for patient safety culture [17]. The present study used SAQ because of its greater adaptation, validation, and international acceptance [21, 22]. In addition, SAQ provides relevance to Saudi culture and comparative analysis of dentists’ safety scores in a similar context [17, 20]. The Agency for Healthcare Research and Quality developed SAQ for safety culture in healthcare settings [3]. There are 36 items in the SAQ instrument which are categorized into six domains: teamwork climate (6 six items), job satisfaction (5 items), perception of management (6 items), stress recognition (4 items), safety climate (8 items), and working conditions (7 items). A five-point Likert scale (Strongly Disagree, Disagree, Neutral, Agree, Strongly Agree) is used for each item of the SAQ. Previous studies confirmed high internal consistency reliability (Cronbach’s alpha α = 091) for the total scale and validity (confirmatory factor analysis) of the SAQ [22, 23]. The score of the SAQ ranges from 0 to 100 and a cut-off ≥ 75 is considered a positive attitude of patient safety. A score of 0 is given to strongly disagree, 25 to disagree, 50 to neutral, 75 to agree, and 100 to strongly agree options [24, 25]. 

### Variables description

Patient safety culture of dental professionals is a dependent variable in the study. Independent variables included age, gender, years of experience in dental practice, type of practice (public and private), level of qualification (general practitioner (GP), specialist, consultant), and qualification obtained from Saudi Arabia or abroad.

### Procedure and ethics

The Deanship of Scientific Research approved the project (IRB 2022 − 343). Permission was obtained from the administrators of hospitals/dental clinics to distribute the questionnaires among dental practitioners who filled the hardcopies of self-administered questionnaires. The questionnaires were collected if dentists provided their responses in the first visits. In case dentists were unable to fill out the questionnaires on the same day of the visit, then they were contacted after one week. A maximum of three visits were made to obtain an adequate response rate from dental professionals. The consent form was designed to provide a brief explanation of the study and participants’ rights of voluntary participation, privacy, and confidentiality. Participants were assured that the collected data were for research purposes only. The study was conducted in accordance with the ethical principles of the Helsinki Declaration.

### Statistical analysis

Data analysis was performed by using SPSS software (IBM SPSS Statistics for Windows, version 22.0. Armonk, NY: IBM Corp). Frequencies, proportions, means, and standard deviations were calculated for different variables of the study. Independent t-test (due to unknown population standard deviation) was performed to compare SAQ scores in two categories of participants. Bivariate and multiple logistic regression analyses (assumptions for multiple logistic regression analysis were: linearity, no outliers, independence, and no multicollinearity) were used to investigate the relationship between positive safety culture and study variables. A *P* < 0.05 was used for statistical significance.

## Results

The questionnaires were distributed among 334 dental professionals; however, 271 participants returned completed questionnaires and the response rate of the study was 78.77%. There were 53.9% of males and 46.1% of females in the study with a mean age of 35.56 ± 6.87 years. About 47.2% of Saudis, 51.7% of general practitioners (GPs), 61.6% of participants with ≥ 10 years of practice, 70.1% of married, and 85.2% from the private practice participated in the study. Almost half of the participants (52%) attended a course of patient safety and 22.1% experienced medical error in the last month. In regards to patient safety score comparing different study variables, it was significantly related to marital status (*P* = 0.041), attendance of patient safety course (*P* < 0.001), and experience of medical error during the last month (*P* = 0.008) (Table 1).

**Table 1:** Demographic data of dental professionals with patient safety score comparison

Study Variables	*N* (%)	SAQ Score Mean ± SD	*P*-value
**Gender**			
Male Female	146 (53.9) 125 (46.1)	66.05 **±** 12.35 64.08 **±** 13.75	0.216
**Nationality**			
Saudi Non Saudi	128 (47.2) 143 (52.8)	63.56 **±** 13.21 66.56 **±** 12.75	0.058
**Place of work**			
Government sector Private sector	40 (14.8) 231 (85.2)	65.33 **±** 12.82 65.11 **±** 13.09	0.922
**Qualifications**			
General practitioner (GP) Specialist/consultant	140 (51.7) 131 (48.3)	65.08 **±** 12.51 65.21 **±** 13.61	0.934
**Qualification obtained from**			
Saudi Arabia Abroad	93 (34.3) 178 (65.7)	63.85 **±** 13.65 65.82 **±** 12.68	0.240
**Income (Saudi Riyal)**			
Less than 10,000 Equal or more than 10,000	70 (25.8) 201 (74.2)	63.06 **±** 13.41 65.87 **±** 12.85	0.120
**Marital Status**			
Single Married	81 (29.9) 190 (70.1)	62.67 **±** 14.06 66.2 **±** 12.46	0.041
**Years in dental practice**			
Less than 10 years Equal or more than 10 years	104 (38.4) 167 (61.6)	64.19 **±** 12.69 65.74 **±** 13.24	0.341
**Attended a patient safety course**			
Yes No	141 (52.0) 130 (48.0)	69.30 **±** 12.71 60.64 **±** 11.86	< 0.001
**Experienced “medical error” during the last month**			
Yes No	60 (22.1) 211 (77.9)	61.19 **±** 13.64 66.27 **±** 12.66	0.008

Descriptive statistics of SAQ and its domains are shown in Table 2. The mean score of the SAQ of the sample was 65.14 ± 13.03. The job satisfaction domain showed the highest mean score (73.27 ± 20.11), followed by the safety climate domain (67.69 ± 16.68), and the working conditions domain (66.51 ± 20.43). However, the lowest mean score was observed for the stress recognition domain (49.58 ± 13.92).

**Table 2:** Descriptive statistics of SAQ and its domains

Domains of SAQ	Range (min-max)	Mean	Standard deviation
Teamwork climate (6 items)	83.33 (16.67–100)	65.87	15.54
Safety climate (7 items)	82.14 (17.86–100)	67.69	16.68
Job satisfaction (5 items)	80.00 (20.00-100)	73.27	20.11
Stress recognition (4 items)	62.50 (18.75–81.25)	49.58	13.92
Recognition of management (5 items)	100 (0-100)	64.52	19.74
Working condition (4 items)	93.75 (6.25–100)	66.51	20.43
Total SAQ	63.89 (27.78–91.67)	65.14	13.03

In the study, 22.5% of participants demonstrated a positive safety culture (≥ 75 SAQ score). A cut-off ≥ 75 is considered a positive attitude of patient safety. In bivariate analysis, the participants who attended a patient safety course were 4.64 times more likely to demonstrate positive safety culture (*P* < 0.001). The attendance of patient safety course remained a statistically significant predictor of positive safety culture among study participants (*P* < 0.001) after controlling for gender, nationality, place of work, qualifications, income, marital status, years in dental practice, and experiencing medical error (Table 3).

**Table 3:** Relationship of demographic data with positive safety culture among dental professionals

Study Variables	Positive Safety Culture Unadjusted Odd Ratio (95% CI)	*P*-value	Positive Safety Culture Adjusted Odd Ratio (95% CI)	*P*-value
**Gender**				
Male Female	1.20 (0.67, 2.13)	0.533	1.23 (0.64, 2.36)	0.534
**Nationality**				
Saudi Non Saudi	0.79 (0.44, 1.40)	0.413	0.77 (0.31, 1.88)	0.563
**Place of work**				
Government sector Private sector	1.0 (0.45, 2.23)	0.999	0.57 (0.21, 1.57)	0.279
**Qualifications**				
General practitioner (GP) Specialist/consultant	0.74 (0.42, 1.31)	0.307	0.91 (0.43, 1.93)	0.811
**Qualification obtained from** Saudi Arabia Abroad	0.91 (0.50, 1.68)	0.775	1.82 (0.68, 4.89)	0.233
**Income (Saudi Riyal)**				
Less than 10,000 Equal or more than 10,000	0.64 (0.32, 1.29)	0.212	0.64 (0.27, 1.53)	0.315
**Marital Status**				
Single Married	0.88 (0.47, 1.66)	0.695	1.261 (0.59, 2.70)	0.551
**Years in dental practice**				
Less than 10 years Equal or more than 10 years	0.60 (0.33, 1.12)	0.106	0.83 (0.19, 3.67)	0.810
**Attended a patient safety course**				
Yes No	4.64 (2.38, 9.08)	< 0.001	4.64 (2.38, 9.08)	< 0.001
**Experienced “medical error” during the last month**				
Yes No	0.54 (0.25, 1.17)	0.114	0.73 (0.32, 1.69)	0.465

The participants who attended a patient safety course demonstrated significantly higher score of teamwork climate domain than those who did not attend a safety course (*P* = 0.001). Similarly, statistically significant relationship of attendance of safety course with safety climate domain (*P* < 0.001), job satisfaction domain (*P* = 0.001), recognition of management domain (*P* < 0.001), and working conditions domain (*P* < 0.001) was observed in the study (Table 4).

**Table 4:** Relationship of attendance of safety course with domains of SAQ

Domains of SAQ	Attendance of safety course Mean ± SD	No attendance of safety course Mean ± SD	*P*-value
Teamwork climate	68.97 **±** 15.71	62.51 **±** 14.69	0.001
Safety climate	73.10 **±** 15.11	61.82 **±** 16.38	< 0.001
Job satisfaction	77.02 **±** 20.47	69.21 **±** 18.97	0.001
Stress recognition	50.53 **±** 13.888	48.56 **±** 13.93	0.244
Recognition of management	68.69 **±** 18.91	59.69 **±** 19.62	< 0.001
Working condition	71.9 **±** 19.66	60.67 **±** 19.69	< 0.001

## Discussion

This study evaluated patient safety culture among dental professionals working in the Eastern province of Saudi Arabia. Patient safety score was significantly related to experience of medical error during the last month. Several studies have explored patient safety culture and its impact on medical errors. Wami et al. found that contributing to patient safety programs improved the patient safety culture score, indicating a potential association between proactive safety initiatives and improved safety culture. [26] Additionally, El-Jardali et al. observed a rise in medical errors reported by respondents, potentially linked to frustration with hospital regulations or increased staff awareness of safety issues [27]. While Şantaş et al. reported a connection between patient safety culture scores and a lower incidence rate of medical errors as reported by nurses, suggesting a potential inverse relationship between safety culture and medical errors [28]. Furthermore, Mekonnen et al. found that the overall patient safety score and most dimension scores were lower than benchmark scores, indicating potential areas of concern related to patient safety culture and its impact on medical errors [29]. Furthermore, Jang et al. observed a positive correlation between perceived patient safety culture and the rate of proper medication mistake reporting among early-career nurses, indicating that safety culture may have a beneficial effect on error reporting practices [30]. Moreover, Mortazavi et al. found poor communication between patient safety atmosphere and medical errors in hospitals, indicating potential gaps in safety culture that may contribute to medical errors. [31]

In our study, 22.5% of participants demonstrated a positive safety culture (≥ 75 SAQ score). There is a growing focus on developing a positive patient safety culture in dentistry. Studies such as those by Bailey & Dungarwalla, Ramoni et al., and Yansane et al. emphasize the importance of teaching, training, and awareness in cultivating a robust patient safety culture within dental practices. [32]Additionally, research by Cheng et al. and Arbianti highlights the positive attitudes and efforts toward understanding and improving patient safety culture among dental healthcare workers. [17, 33] Furthermore, studies by Choi et al., Palmer et al., and Abdelnaby et al. demonstrate the positive attitudes of dental professionals and students towards patient safety culture and the willingness to comply with patient safety guidelines [34–36]. The research by Tagar and Yamalik & Dijk also underscore the importance of increasing awareness and understanding of patient safety culture among dental practitioners [37, 38]. Overall, these studies underscore the importance of promoting a positive patient safety culture in dentistry through education, training, and increased awareness. These efforts are essential in fostering a culture of safety, improving patient care outcomes, and minimizing adverse events in dental practice.

Positive patient safety culture was not associated with the gender of the dental practitioner in our study. Similarly, gender was not associated with patient safety factors in Dutch GP cooperatives [39]. In addition, Khoshakhlagh et al. reported that demographic factors, including gender, did not significantly affect the level of patient safety culture in public and private hospitals in Iran [40]. This was contrary to other studies that identified gender as a contributing factor to the variance in patient safety culture in Indonesia and China [41, 42]. Marital status was associated with a positive patient safety culture in this study. Interestingly, marital status has been found to have implications for patient safety culture and healthcare professionals. Studies have shown that sociodemographic factors, including marital status, influence safety culture in healthcare settings [43, 44]. Furthermore, the influence of patient safety attitudes on turnover intentions among new nurses in China suggests a potential interplay between marital status, patient safety culture, and professional identity [45]. 

In this study, positive patient safety culture was not associated with the qualification of dental practitioners (GPs vs. Specialists or Consultants). While in other studies, the level of qualification has been associated with dentists’ attitudes and practices, such as the use of personal protective equipment (PPE) during the COVID-19 pandemic [46, 47]. Recent graduates and those with postgraduate degrees demonstrated higher levels of practice on PPE compared to others [47]. Additionally, positive patient safety culture was not associated with years of experience in this study. This was contrary to a Korean study that assessed dentists’ perceptions and attitudes concerning radiation safety and protection that have been influenced by their years of experience, with those having less than 10 years of experience gaining awareness of radiation safety concepts through various pathways, including school education and radiation safety officers [48]. 

To ensure patient safety, it is crucial for dentists to undergo appropriate training and continuing professional development [49]. This will support safe clinical practice by sustaining skills and keeping dentists up-to-date [50]. Dentists’ practices and patient safety are closely linked, and training and work environment significantly impact the frequency of safety practices in dental offices [51]. Additionally, dentists’ perception of medication safety and their practice significantly impact patient safety [52]. Furthermore, specialized training on infection control, drug administration in conscious sedation and occupational safety is essential for all practicing dentists and dental students to prevent injury and infection in dental clinics [53, 54]. 

In addition to training, it is essential for dentists to adopt new technologies and adhere to safety standards to ensure patient safety. Adoption of new technologies by dental practitioners is crucial, but concerns about patient safety when using certain technologies indicate the need for thorough training and awareness of safety implications [55]. Furthermore, dentists should follow all guidelines and safety precautions to avoid deleterious effects, emphasizing the importance of strict adherence to safety protocols in dental practice [56]. 

The present study provided valuable information about patient safety culture perceptions among dentists with adequate response rate, and data may be used to guide the planning and organization of continuing education programs for dentists to provide safer dental care and prevent adverse events. However, there are several limitations in this study. The sample being mainly from the private sector (85.2%) might be considered a limitation, as the sample needs to be more representative for both public and private sectors. The second limitation could be the lack of a probability sampling technique. This may affect the generalizability of results to the whole dentist population in the Eastern Province of Saudi Arabia. In addition, we were limited by dental practitioners’ behaviors, such as error reporting and adverse event reduction indicating the potential impact of safety culture on clinical outcomes. Future studies should aim to investigate the reasons for differences in patient safety culture as well as to focus on evaluating the efficacy of interventions aimed at improving safety culture. Furthermore, patient-based outcomes and perceptions of dental care might be worth investigating and correlated to the patient safety culture of health care providers.

## Conclusion

In our study, patient safety culture was significantly related to the attendance of safety courses, marital status, and experiencing medical error. About one out of four dental professionals demonstrated a positive attitude towards patient safety culture which was significantly associated with the attendance of the safety course.

## Data Availability

Data is provided within the manuscript .
